# Whole-Genome Sequence of a Clinical Enterococcus gallinarum Strain in a Positive Hemoculture from Siriraj Hospital, Thailand

**DOI:** 10.1128/mra.00605-22

**Published:** 2022-11-01

**Authors:** Thunchanok Yaikhan, Iyarace Kampakdee, Piroon Jenjaroenpun, Iyarit Thaipisuttikul, Thidathip Wongsurawat, Witchuda Kamolvit

**Affiliations:** a Department of Microbiology, Faculty of Medicine, Siriraj Hospital, Mahidol University, Bangkok, Thailand; b Division of Bioinformatics and Data Management for Research, Research Group and Research Network Division, Research Department, Faculty of Medicine, Siriraj Hospital, Mahidol University, Bangkok, Thailand; c Siriraj Long-read Lab, Faculty of Medicine, Siriraj Hospital, Mahidol University, Bangkok, Thailand; Loyola University Chicago

## Abstract

Enterococcus gallinarum is a rare causative agent of hospital-acquired bacteremia. Here, we reported the complete genome of E. gallinarum strain WKB01, which was directly isolated from a positive hemoculture in Siriraj Hospital, Thailand. Comprehensive analysis demonstrated the clinically relevant *tetM* gene presenting in its genome.

## ANNOUNCEMENT

Enterococcus gallinarum is a Gram-positive facultatively anaerobic bacterium that is typically isolated from poultry intestinal tracts (1). It is not commonly found in human clinical specimens and is rarely associated with human disease. However, it can cause nosocomial infection, especially in immunocompromised individuals (2). Several studies reported the increasing incidence of E. gallinarum related to bacteremia and septicemia (3, 4). In this work, a positive hemoculture result was obtained from the microbiology laboratory of Siriraj Hospital (Bangkok, Thailand). After the BacT/Alert hemoculture system alerted regarding the positive result, Gram staining and subculture on solid agar were immediately performed. An isolated colony was collected for identification using the Vitek2 system (5). Another 200 μL of blood was collected from the bottle and processed with the BIOFIRE blood culture identification 2 (BCID2) panel (bioMérieux, USA) (6). The positive hemoculture was identified to contain *Enterococcus* spp. by the Vitek2 system, whereas the BCID2 panel was not able to detect the existence of bacteria and antimicrobial resistance genes in the sample.

To rapidly identify bacteria in the positive blood culture without primary growth, 15 mL of the blood culture was taken directly from the bottle, and then human cell depletion was performed using low-speed centrifugation at 1,600 rpm for 10 min. The supernatant, containing bacterial cells, was collected. The ZymoBIOMICS DNA miniprep kit (Zymo Research, USA) was used for bacterial DNA extraction; the extraction protocol was according to the manufacturer’s instructions (7, 8). The purified bacterial DNA was further used for genome sequencing with a long-read Oxford Nanopore Technologies (ONT) system. A DNA library was generated using a rapid sequencing kit (SQK-RAD004; ONT) and loaded into a Flongle flow cell v9.4.1 (FLO-MIN106). Sequencing was performed with a MinION Mk1B sequencer with an adapter. Guppy v6.0.1 with the super accuracy (SUP) model was applied for demultiplexing of base-called signals from the Nanopore sequencer (9). ONT raw reads were filtered based on a mean quality score of 8, using NanoFilt v2.5.0. The reads with >1,000 bases were employed for *de novo* assembly and whole-genome sequence construction with Flye v2.8.3 (10), followed by an error correction step that included two rounds of polishing with Racon v1.3.3 (https://github.com/isovic/racon) and one round of polishing with medaka v0.6.5 (https://github.com/nanoporetech/medaka). Default parameters were used for all software unless otherwise specified. The *de novo* assembly generated 4 contigs, with genome coverage of about 132×. The long-read *N*_50_ value was 7,222 bp. The longest contig (GenBank accession number JALBCZ010000001) was suggested to be a circular contig, with a size of 3,357,821 bp, by Flye and was classified as Enterococcus gallinarum by GTDB-Tk (11), using an average nucleotide identity (ANI) cutoff value of 95% for the species boundary. The 3 linear contigs (GenBank accession numbers JALBCZ010000002, JALBCZ010000003, and JALBCZ010000004) are highly similar to a plasmid from Enterococcus casseliflavus (GenBank accession number CP046124.1), with ~99% sequence identity using the BLASTn database. The assembly and gene information are presented in Table 1.

**TABLE 1:** Genome characteristics of the Enterococcus gallinarum strain collected from a positive hemoculture by the microbiology laboratory of Siriraj Hospital, Thailand

Parameter	Finding
Total sequence length (bp)	3,364,430
No. of contigs	4.0
Longest sequence size (bp)	3,357,821
*N*_50_ (bp)	7,222
Genome coverage (×)	132
GC content (%)	40.4
No. of coding sequences	3,485.0
Avg protein length (amino acids)	240.0
Coding proportion (%)	76.64
No. of rRNAs	80.0
No. of tRNAs	61.0
No. of CRISPR arrays	0.0
FastANI ANI (GTDB-Tk) (%)	97.27
FastANI taxonomy (GTDB-Tk)	Kingdom *Bacteria*, phylum *Firmicutes*, class *Bacilli*, order *Lactobacillales*, family *Enterococcaceae*, genus *Enterococcus*, species *Enterococcus gallinarum*
FastANI alignment fraction (GTDB-Tk)	0.83
GenBank assembly accession no. for closest placement reference (GTDB-Tk)	GCF_001544275.1
Closest placement radius (GTDB-Tk) (%)	95

This clinical research study was permitted by the Siriraj Hospital institutional review board under certificate of approval Si964/2021.

### Data availability.

The complete genome sequence has been deposited in GenBank under BioProject accession number PRJNA813694, BioSample accession number SAMN26289057, SRA accession number SRR19138402, and genome accession number JALBCZ010000000.
